# Phenotypic divergence of sand flathead (*Platycephalus bassensis)* between heavily and lightly fished regions in Tasmania, Australia

**DOI:** 10.1093/conphys/coag001

**Published:** 2026-02-05

**Authors:** Harriet R Goodrich, Finlay Rossiter-Hill, Asta Audzijonyte, Barrett W Wolfe, Rachel Breslin, Sean R Tracey

**Affiliations:** Institute for Marine and Antarctic Studies, University of Tasmania, Private Bag 49, Hobart, TAS 7001, Australia; Institute for Marine and Antarctic Studies, University of Tasmania, Private Bag 49, Hobart, TAS 7001, Australia; Institute for Marine and Antarctic Studies, University of Tasmania, Private Bag 49, Hobart, TAS 7001, Australia; Institute for Marine and Antarctic Studies, University of Tasmania, Private Bag 49, Hobart, TAS 7001, Australia; Institute for Marine and Antarctic Studies, University of Tasmania, Private Bag 49, Hobart, TAS 7001, Australia; Institute for Marine and Antarctic Studies, University of Tasmania, Private Bag 49, Hobart, TAS 7001, Australia

**Keywords:** Boldness, fisheries induced evolution, fishing pressure, metabolism, recreational angling

## Abstract

Fishing is one of the most sustained forms of human–wildlife interaction and can alter trait distributions through selective harvest and repeated disturbance. Such changes, whether plastic or evolutionary, may alter productivity, resilience, and recovery in exploited species. The sand flathead (*Platycephalus bassensis*), a benthic ambush predator with strong site fidelity, supports lutruwita (Tasmania’s) largest recreational fishery and is exposed to contrasting levels of fishing pressure across its range. In southern Tasmania, fishing mortality exceeds natural mortality more than fivefold and biomass has fallen below 20% of unfished levels, while northern regions remain comparatively lightly fished. This regional contrast offers a natural setting in which to investigate whether sustained harvest is associated with regional differences in physiology and behaviour, and whether such variation is more consistent with fishing pressure, environmental conditions, or their interaction. We compared mass-specific metabolic rate, boldness, and size-at-age between sand flathead from heavily and lightly fished regions. Metabolic rate was measured using intermittent flow-through respirometry, and boldness was quantified in a shuttlebox based on exploration latency and bait strikes. Fish from the heavily fished south exhibited smaller size-at-age, a 62% higher mean metabolic rate, and a transient post-capture elevated metabolic rate consistent with greater metabolic reactivity or stress responsiveness, whereas boldness did not differ between regions. Our findings align with other exploited systems and raise the possibility that trait diversity of sand flathead in southern regions of Tasmania have been shaped, at least in part, by fisheries selection. We discuss the relevance of these results for fisheries management and emphasize the importance of assessing trait variation in wild populations, where expression is likely shaped by the interactive effects of fishing pressure and local ecological conditions.

## Introduction

Humans are now recognized as one of the strongest evolutionary forces on Earth, particularly in aquatic systems where fishing mortality exceeds natural mortality (Heino et al., 2015). In exploited fish populations, this elevated mortality can drive shifts in life-history traits, selecting for earlier maturation and increased reproductive investment, even in the absence of size-selective harvest (Jørgensen et al., 2007; Audzijonyte et al., 2013; Clark et al., 2017). When larger individuals are disproportionately removed, as is common in many fisheries, selection further favours smaller size-at-age and accelerated life-history strategies (Conover and Munch, 2002; Conover et al., 2005). These patterns, collectively referred to as fisheries-induced evolution, can lead to reduced somatic growth, smaller adult body size and greater reproductive allocation (Swain et al., 2007; Audzijonyte et al., 2013; Waples and Audzijonyte, 2016). While such changes may confer short-term fitness advantages under high mortality, they often reduce long-term productivity, limit recovery following reduced fishing mortality and constrain resilience to environmental change (Jørgensen et al., 2007; Audzijonyte et al., 2015; Clark et al., 2017).

For harvest-based selection to act, there must be consistent phenotypic variation among individuals in their vulnerability to capture. Vulnerability typically reflects a combination of life history, morphological, physiological and behavioural traits that together determine whether and how a fish encounters, responds to and evades fishing gear (Biro and Post, 2008; Arlinghaus et al., 2017). Traits associated with bioenergetics and behaviour, such as metabolic rate (MR) and boldness, are therefore increasingly recognized as important targets of fishing selection (Killen et al., 2015; Klefoth et al., 2017; Hollins et al., 2018). Individuals with high standard metabolic rates (SMR) often forage more actively and grow faster, but this elevated activity can increase capture risk (Stamps, 2007; Biro and Post, 2008; Hessenauer et al., 2015; Killen et al., 2015; Binder et al., 2016; Väätäinen et al., 2018). These same individuals also tend to show stronger stress responses and slower recovery following disturbance (Careau et al., 2008; Killen et al., 2011; Tudorache et al., 2013; Metcalfe et al., 2016; Yuan et al., 2018). The selective removal of such high MR phenotypes can lead to populations dominated by low-MR and more cautious behaviour, a pattern sometimes referred to as an exploitation-induced ‘timidity syndrome’ (Arlinghaus et al., 2017; Klefoth et al., 2017). Many exploited species therefore show trends consistent with selection against high-performance, energetically costly phenotypes (Biro and Post, 2008; Hessenauer et al., 2015). However, the direction and strength of these effects depend on species traits, gear selectivity, and ecological context (Wilson et al., 2011).

When fishing mortality is extremely high and acts before reproduction, the selective landscape may shift. Rather than favouring the persistence of slow, cautious individuals, strong harvest might instead favour fast-developing individuals that grow and mature early. Such patterns are predicted when elevated mortality reduces the fitness value of delayed reproduction, thereby favouring faster life histories (Heino et al., 2015). Under these conditions, traits that accelerate growth and reproduction, such as higher MR could be favoured (Pettersen et al., 2016). This scenario has been proposed in systems subject to intense harvest, where rapid development and early maturation evolve as adaptive responses to high fishing mortality (Conover and Munch, 2002; Conover et al., 2005; Audzijonyte et al., 2013). Indeed, early maturation can lead to growth stunting, a pattern commonly reported among exploited fish stocks where energy is diverted from somatic growth to reproduction (Matsumura et al., 2011; Han et al., 2025). Although, fishing can also create feedbacks through density-dependent processes. For example, if biomass declines with sustained exploitation, reduced competition and greater resource availability may relax selection against energetically costly phenotypes, allowing individuals with higher MR to persist (Crespel et al., 2021). In contrast, if food becomes scarce or habitat quality declines, energetic constraints may favour slower, more efficient phenotypes. The balance between all of these processes can shape both the average phenotype of a population and the extent of among-individual variation, yet remains poorly understood in natural systems where experimental control is not always possible.

The southern sand flathead (*Platycephalus bassensis*) provides a valuable model for exploring how sustained fishing pressure may influence physiological and behavioural traits in the wild. This demersal ambush predator is among lutruwita/Tasmania’s most important recreational species, targeted by more than 70% of active fishers (IMAS, 2020; Department of Natural Resources and Environment Tasmania, 2023). Fishing intensity, however, varies strongly across the state. In southern Tasmania, sand flathead experience fishing mortality more than fivefold greater than natural mortality (fishing mortality (F) ≈ 1.47 yr^−1^, natural mortality (M) ≈ 0.28 yr^−1^), and biomass is now less than 20% of unfished levels (Krueck et al., 2023; Tracey and Stark, 2024). In contrast, northern sand flathead are lightly exploited and remain relatively stable. Regional differences in the size structure and growth of sand flathead (Krueck et al., 2023) suggest that sustained harvest could be shaping the phenotypic composition of local populations.

This study therefore takes advantage of this contrast in harvest intensity to examine whether long term variation in recreational fishing pressure on sand flathead in Tasmania is associated with measurable differences in metabolism and behaviour, and whether these patterns are most consistent with fishing driven selection, environmental plasticity, or their interaction. To address this, we measured boldness, post-capture oxygen consumption, metabolic rate (MR), and size-at-age in sand flathead from both southern Tasmania, where fishing pressure is high, and from the comparatively lightly fished northeast (herein ‘north’). We tested two contrasting hypotheses. First, if fishing selectively removes bold individuals with high MR, southern fish should exhibit reduced boldness, lower MR, and slower growth, consistent with a more energy efficient and risk averse phenotype. Alternatively, strong harvest mortality in the south may favour traits associated with a faster pace of life, resulting in elevated MR, smaller size at age, and heightened stress responsiveness.

## Materials and methods

All research was carried out following approval from the University of Tasmania Animal Ethics Committee (project number: AEC 00030759) and collection of sand flathead (*Platycephalus bassensis*) was conducted under Department of Natural Resources and Environment Tasmania (DNRET) Scientific Research Permit 23 087.

### Study system and species profile

Sand flathead (*Platycephalus bassensis*) is a demersal, site-attached ambush predator common in shallow coastal waters of Tasmania (Tracey et al., 2020). It supports the state’s largest recreational fishery and is targeted by more than 70% of active recreational fishers (IMAS, 2020; Department of Natural Resources and Environment Tasmania, 2023; Krueck et al., 2024; Tracey and Stark, 2024). Recent statewide surveys estimate that approximately 1.08 million sand flathead were caught in 2023, accounting for around half of all recreational finfish captured by number. Of these, 381 000 fish were retained and 643 000 (63%) were released, largely because individuals were below the legal size limit (Tracey and Stark, 2024). Although many fish are released, nearly all legal-sized individuals are retained for consumption, contributing to an estimated annual fishing mortality (F) of 1.47 yr^−1^, equivalent to the removal of ~ 93% of adult females in heavily fished regions (Krueck et al., 2023).

This strong regional gradient in fishing pressure provided the basis for the present study. The southern site, North-West Bay (*n* = 92; −43.076, 147.310), located in the D’Entrecasteaux Channel, southern Tasmania, represents a heavily exploited region with high local effort and a history of sustained harvest. Indeed, statewide slot and bag limits introduced by the Tasmanian Government in 2023 were implemented to promote recovery of depleted sand flathead stocks in the south. The new regulations set a slot limit of 35–40 cm across most state waters, protecting both immature fish and large, highly fecund adults. At King and Flinders Islands (Northern Tasmania), only the 35 cm minimum applies, reflecting the non-depleted status of those regions. Bag and on-water possession limits were also adjusted to reflect regional differences in stock status, with a daily limit of two fish in the southern D’Entrecasteaux Channel and Derwent region, five fish along the broader eastern and south-eastern coasts, and ten fish across northern and western waters where stocks remain less impacted.

**Figure 1:**
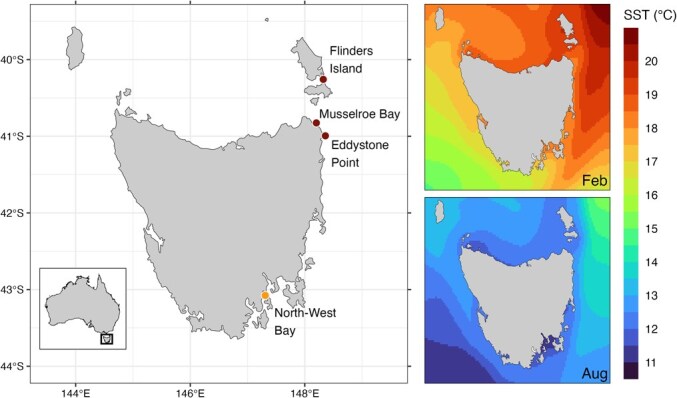
Approximate locations and coordinates of sampling sites for sand flathead(*Platycephalus bassensis*) in northern and southern Tasmania. Lightly fished northern sites (redmarkers) included Flinders Island (−40.258, 148.323), Eddystone Point (−40.994, 148.360),and Musselroe Bay (−40.826, 148.202) (27.71 ± 0.19 cm; 137.78 ± 3.14 g; n = 80). The heavilyfished southern site (yellow marker) was North-West Bay within the D’Entrecasteaux Channel(−43.076, 147.310) (23.11 ± 0.23 cm; 81.67 ± 2.83 g; n = 92). Mean SST panels (right) show10-year monthly climatologies for February and August (2014–2024) derived from GHRSST. Geo-Polar Blended SST Analysis dataset (OSPO 2015). Data were averaged across 0.04°spatial grids (143.5–149.5° E; −44–−39.5° S), and plotted with a viridis colour scale. Warmerconditions occur in late summer (February) and cooler conditions in winter (August), but mean SST differences between northern and southern sites are small (< 3°C).

For the current study, northern sampling sites included coastal waters off Flinders Island (−40.258, 148.323), Eddystone Point (−40.994, 148.360), and Musselroe Bay (−40.826, 148.202) (*n* = 82) (Fig. 1). These areas are characterized by low fishing pressure, low human population density, and greater ocean exposure that limit boating access and fishing effort. Statewide surveys support this spatial pattern, showing that over half (57%) of the total statewide catch of sand flathead are taken from southeast Tasmania, while only 19% are taken from the north of the state (Tracey and Stark, 2024). These sites were therefore selected to reflect pronounced contrasts in fishing intensity and observed differences in population size and age structure, providing a strong basis to assess potential regional divergence in physiological traits (Fig. 1). Both regions lie within Tasmania’s temperate eastern bioregion and experience similar annual temperature regimes typical of the southeastern coastal waters (Fig. 1) (Ridgway, 2007; Ridgway and Ling, 2023). Both regions encompass the sandy inner-shelf habitats typical of sand flathead with larvae and adults concentrated in nearshore sand and mixed sand–seagrass environments that support prey assemblages dominated by small fishes and crustaceans (Ayling et al., 1975; Jordan, 2001).

### Capture and transport

Sand flathead (*n* = 174, see Table 1 for morphometric data) were captured from a boat using standardized hook and line-fishing techniques comprising a paternoster rig with two small circle hooks, with the aim of collecting individuals of comparable size across sampling regions. Both hooks were baited with squid or chicken soaked in tuna oil. Fishing commenced upon arrival at randomly selected marks within suitable habitats at each site, with the vessel allowed to drift naturally across the area. If the boat drifted off target, it returned to the location to repeat the process. Upon capture, fish were dehooked using a standard de-hooking tool, and only individuals in good condition, free of abrasions, signs of disease, deformities and hook injuries were retained for further study. Although sand flathead can exceed 45 cm in total length (TL), individuals selected for this study ranged from 17.0 to 30.5 cm TL. Age was determined retrospectively from otolith analyses (described below), which showed that these fish spanned 1–8 years (Table 1). Angling was selected as the capture method because it is virtually the only method used in the Tasmanian recreational fishery and thus is the means by which harvest-related selection would act on sand flathead traits (Fraser et al., 2021; Krueck et al., 2024; Tracey and Stark, 2024). While catch per unit effort (CPUE) was not determined for sampling in this study, standardized catch rates indicate that encounter probability and overall abundance are comparable among regions, with differences confined to the proportion of large fish (>320 mm), consistent with size truncation among heavily fished southern regions (Fraser et al., 2021; Krueck et al., 2023, 2024). Likewise, spatial distributions show equivalent catch rates across northern and southern regions in recent years, suggesting similar fish density and availability (Fraser et al., 2021). These data suggest that sampling was not regionally biased by capture vulnerability.

**Table 1:** Morphometric summary statistics for sand flathead (*Platycephalus bassensis)* captured from the southern and northern Tasmania in 2024

	Total length (cm)		Total mass (g)		Body condition (K)		Age (yrs)	
	Mean ± S.E.M	Range (min—max)	Mean ± S.E.M	Range (min—max)	Mean ± S.E.M	Range (min—max)	Mean ± S.E.M	Range (min—max)
South (*n* = 92)	23.11 ± 0.23	17.0–28.0	81.67 ± 2.83	28.28–154.6	0.64 ± 0.01	0.50–0.81	4.17 ± 0.19 (n = 76)	1–8
North (*n* = 82)	27.71 ± 0.19	21.5–30.5	137.78 ± 3.14	55.36–190.5	0.63 ± 0.01	0.44–0.76	2.7 ± 0.14 (n = 68)	1–6
Respirometry South (*n* = 12	22.75 ± 0.60	18.0–26.0	75.32 ± 6.18	29.27–116.60	0.62 ± 0.02	0.50–0.67	4.2 ± 2.2 (n = 10)	1–8
Respirometry North (*n* = 8)	26.81 ± 0.65	23.5–29.0	109.12 ± 7.02	72.87–133.67	0.51 ± 0.001	0.51–0.59	2.0 ± 0.7 (*n* = 7)	1–3

Values are presented as mean ± standard error of the mean (S.E.M). Northern data represent pooled samples from Flinders Island, Musselroe Bay, and Eddystone Point. ‘Respirometry south’ and ‘Respirometry north’ refer to individuals selected for the respirometry analyses described in Section 2.5. Sample sizes for age differ from whole population due to poor readability for analyses of some otoliths. Metrics include total length (mm), total mass (g), Fulton’s condition factor (K) and age (yrs).

Upon capture, fish were temporarily housed in aerated, temperature-monitored seawater tubs at a density not exceeding 25 kg/m^3^ (approximately 6–8 fish per 70 L tub). Dissolved oxygen was maintained at a target minimum 90% air saturation, and water temperature at 13 ± 5°C. While on board, seawater was frequently exchanged to maintain water quality. Upon return to the launch jetty, 90% of the water in each holding tub was replaced with fresh seawater prior to transport. Tubs were then placed in the tray of a Ford F250 Ute, covered with secure lids and a hard roll-up tray cover to minimize light and heat exposure. Transport duration in the vehicle was approximately 25 minutes from the southern site, and approximately 5 hours from the northern site. During the northern transport, three scheduled stops were made to replenish seawater and monitor dissolved oxygen and temperature. No mortalities occurred during capture or transport.

### Animal husbandry

Following capture and transport, fish were transferred to the Temperate Research Facility at the Institute for Marine and Antarctic Studies, Taroona (TAS, Australia) in September (southern population) and November (northern populations) of 2024. Fish were distributed among thirty 300-L circular tanks integrated into a 10 000-L recirculating aquaculture system, at a density of 6–7 fish per tank per population (equivalent to ~ 42 L per 100 g of biomass). Raw seawater from the Derwent Estuary was continuously supplied to the Temperate Research Facility, where it underwent mechanical filtration (to 50 μm) and UV biological treatment before entering one of three temperature-controlled sumps. Water was then pumped through the system, delivered to tanks via a central manifold, and returned through standpipe overflows for recirculation. Temperature control was maintained using three integrated heater–chiller units (Rheem Australia Pty Ltd, Australia), as well as two inline Optima compact heaters (Electro Engineering Ltd, England). Throughout the trial, water parameters were maintained at 12.5 ± 1°C, pH at 8.11 ± 0.01, NH_3_-N 0.2 ± 0.02 mg l^−1^, NO_2_ 0.4 ± 0.2 mg l^−1^, NO_3_ 0 ± 0 mg l^−1^ and salinity 34.88 ± 1.0 ppt. Surface water temperatures during the southern and northern sampling periods ranged between 10–13°C and 13–16°C, respectively. Fish were maintained on a 13 h light:11 h dark cycle using Fluval Smart LED aquarium lighting. The light schedule included a day setting (6:00–19:00; 5% blue and purple, 0% white light), a night phase (19:00–22:00; 5% blue light), and complete darkness during the sleep phase (22:00–6:00). Fish were fed to satiation once daily using Biomar EFICO Sigma 6-mm sinking pellets. All individuals began feeding within 48 hours of introduction to tank conditions. To track individual performance, fish were implanted with passive integrated transponder (PIT) tags (Biomark, USA) following anaesthesia with 20 mg L^−1^ of Aqui-S®. At the conclusion of all experimental procedures, fish were euthanized with an overdose of Aqui-S® (60 mg L^−1^), after which otoliths were removed by dissection and aged using methods described by Hyndes (1992) and Coulson et al. (2014).

### Size and size at age

To examine regional differences in size and size-at-age, we analyzed publicly available age–length data for sand flathead from the 2024 IMAS Fisheries Independent Survey of recreational fisheries (https://tasfisheriesresearch.org/sfh/surveys/lf/). In this survey, fish were sampled from Flinders Island in the north (*n* = 141) and from Frederick Henry Bay and the D’Entrecasteaux Channel in the south (pooled, *n* = 166). These data are presented in Fig. 3. We prioritized this dataset over our study sample due to the larger sample sizes at each site, but equivalent analyses were also performed on our subsample of fish (Supplementary Fig. S1).

### Repeated measures of metabolic rate

To examine metabolism over time and assess post-capture reactivity, metabolic rate (MR) was measured weekly over a three-week period in a subsample of fish (*n* = 11 south, *n* = 8 north), beginning on Day 3 post-capture. Each measurement represented an estimate of baseline metabolism, reflecting the minimum energy expenditure required to maintain basic physiological function in a resting, undisturbed, and post-absorptive state (c.f. standard metabolic rate; Chabot et al. (2016)), other than any transitory elevation arising from stress of capture event or transfer to captivity. Measures of MR were conducted overnight in complete darkness (4:00 pm—8:00 am), across three timepoints. Days 3–5 (Week 1), Days 10–12 (Week 2), and Days 21–23 (Week 3) post-catch. Fish were grouped in sets of four and assigned to specific days so that the same individuals were measured on the same day of each week (e.g. Fish A–D on Days 3, 10, and 21; Fish E-H on Days 4, 11 and 22). This design allowed for repeated measures of each fish across the three weeks. Prior to each trial, fish were fasted for 48 hours to eliminate the influence of postprandial metabolism (i.e. specific dynamic action) (Secor, 2009; Goodrich et al., 2024).

**Figure 2:**
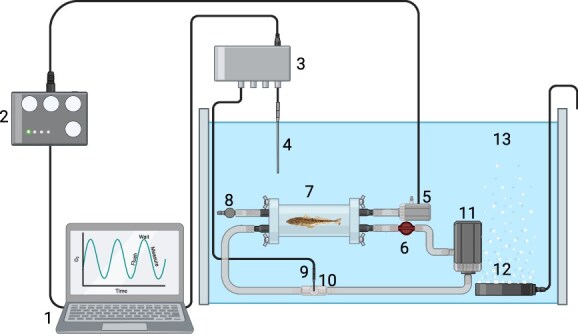
Schematic of the intermittent-flow respirometry system used to measure whole-animal oxygen uptake rate (MO₂) in sand flathead (*Platycephalus bassensis*). The setup includes: (1) a computer running AquaResp 3, Aqua OxyLog, Python 3.3, and Pyro OxyLogger software for data logging and cycle control; (2) an USB-controlled Cleware switch (Cleware GmbH, Germany) automating the flush, wait, and measurement cycles; (3) a 4-channel FireSting oxygen meter (PyroScience GmbH, Germany) recording O₂ content (mg/L); (4) a PyroScience temperature probe; (5) a Tetra WP300 mini pump (Tetra Werke, Germany) for chamber flushing; (6) an adjustable valve regulating flow; (7) individual respirometry chambers containing each fish; (8) a one-way valve ensuring directed water outflow during flushing; (9) an O₂ optode (SPFIB-BARE, PyroScience GmbH, Germany) housed with a flow-through cell (10) (OXFTC, PyroScience GmbH, Germany); (11) an Eheim pump (Eheim GmbH & Co., Germany) circulating water within chambers, through the flow through cell and over the optode; (12) an air stone to maintain oxygen saturation in the water bath and (13) a 300-L water bath/reservoir housing the respirometry chambers. Created in BioRender. Goodrich, H. (2026) https://BioRender.com/ck9qet9

Whole-animal metabolic oxygen consumption rate (MO₂) was assessed as a proxy for aerobic MR using an intermittent-flow respirometry system (Fig. 2). Prior to measurements, each fish (*n* = 11 south, *n* = 8 north) was weighed (south: 79.41 ± 5.90 g; north: 109.12 ± 6.57) and individually placed in a 3.15-L acrylic respirometry chamber (410 mm x 100 mm, Eagle Plastics) for a fish-to-chamber-ratio of 2.5–3.5%. Chambers were submerged within a 300-L aerated seawater bath (35 ppt) maintained at 12 ± 0.5°C using a TECO TK500 chiller (TECO Refrigeration Technologies, Italy), located in a temperature-controlled room (ambient 9 ± 0.5°C). Prior to each trial, chambers were inspected for leaks. After each run, the chambers, tubing, and water bath were drained and cleaned with freshwater and bleach. Fresh seawater (see above for filtration and UV sterilization details of seawater supplied to the Temperate Marine Lab) was then supplied to the bath before the next trial.

Each chamber was fitted with a Loligo respirometry lid and sealed with O-rings. Water was recirculated through the chamber using an Eheim Universal 1046 pump (Eheim GmbH & Co., Germany), passing over an optical oxygen sensor (SPFIB-BARE; PyroScience GmbH, Germany) housed within a flow-through cell (OXFTC; PyroScience GmbH, Germany). Probes were recalibrated to 0% and 100% air saturation at the start of each week of trials, with probe sampling frequency set at every 10 seconds for the duration of each respirometry trial. Oxygen content was recorded in real time using a 4-channel FireSting oxygen meter (PyroScience GmbH, Germany) and logged using Aqua OxyLog software.

The respirometry system operated on an automated flush–wait–measure cycle controlled by AquaResp 3 (Svendsen et al., 2016) and a USB-4 Cleware switch (Cleware GmbH, Germany). A 5-minute flush phase using a Tetra WP300 mini pump (Tetra Werke, Melle, Germany) replenished oxygen levels with fully oxygenated seawater to normoxia (≥90% air sat) in between measurements, followed by the chambers being sealed for a 1-minute wait phase to stabilize flow before a 25-minute measurement phase. Flush flow was regulated using an in-line valve and directed via a one-way valve to prevent backflow. This cycle was repeated continuously overnight, yielding approximately 30–35 measurement cycles per fish.

Mass-specific MO₂ (mg O₂ kg^−1^ h^−1^) was calculated during the sealed measurement phase as the product of the slope of oxygen decline (s; mg O₂ L^−1^ h^−1^) and the effective volume of the chamber (*V*_resp_; L), corrected for fish displacement, divided by the individual’s body mass (m; kg):


$$ {MO}_2=\frac{\kern0.5em s\times{V}_{\mathrm{resp}}}{m} $$

Where:


$$ s=\frac{O_{2\ \mathrm{initial}}-{O}_{2\ \mathrm{final}}}{\mathrm{time}\ \mathrm{initial}-\mathrm{time}\ \mathrm{final}\ } $$

Raw oxygen saturation data were processed using AquaResp 3 (Svendsen et al., 2016, 2019) which converted percent air saturation to oxygen concentration (mg L^−1^) and applied linear regression to estimate the rate of decline in oxygen over time. To ensure reliable and biologically meaningful estimates, only measurement phases with a regression coefficient *R*^2^ ≥ 0.96 were retained. As noted by Chabot et al. (2016), low *R*^2^ values often indicate departures from steady-state conditions due to spontaneous fish activity, poor mixing, or sensor instability, any of which can distort MO₂ estimates. Applying this threshold reduces noise and improves the robustness of MR values by excluding unreliable slopes. While this threshold improves data robustness by filtering out unreliable slopes, it has also been shown to overestimate MR in some cases (Chabot et al., 2021).

Mass-specific MO₂ (mg O₂ kg^−1^ h^−1^) assumes isometric scaling of metabolic rate (i.e. mass scaling exponent *b* = 1). While metabolic rate typically scales allometrically with body mass (*b* < 1 e.g. ~ 0.79 ± 0.11 in fish) (Kleiber, 1947; Clarke and Johnston, 1999), this approach is standard in fish respirometry when fish groups have a limited size range and absence of significant body size differences among individuals used in trials (see Table 1). However, to account for the effects of body size on metabolic rate, we also standardized MO₂ to a common body mass of 1 kg using an allometric scaling exponent of 0.79 (i.e. to mg O₂ kg^-0.79^ h^−1^, see supplementary files) (Clarke and Johnston, 1999; Killen et al., 2010; Norin and Gamperl, 2018). Background oxygen consumption was measured in empty chambers at the end of the trial for 1 h using the same flush wait and measure cycle described above. Background respiration consistently accounted for less than 1% of resting MO₂. Consequently, no background correction was applied to the data.

Baseline MR was estimated from MO₂ measurements with the mean of the lowest normal distribution method (MLND; Chabot et al., 2016). The MLND represents the lowest cluster of MO₂ data points, grouped using model-based normal distribution clustering with the Mclust function of the R package *mclust* (Scrucca et al., 2016). The reliability of this estimate was evaluated using the coefficient of variation (CV_MLND_) which assesses the variability of the lowest normal distribution relative to its mean. High CV_MLND_ values reflect substantial variation across repeated oxygen consumption measurements within a trial. This variation may be due to residual activity in fish, spontaneous movement during measurement phases, or other sources of noise. When CV_MLND_ exceeds the reliability threshold of 5.4% Chabot et al. (2016), the MLND method is considered less robust, and the quantile method (20th percentile of all MO₂ values) is recommended as an alternative estimator of MR. MR values were calculated using the *calcSMR* function in the R package fishMO2, following Chabot et al. (2016).

### Boldness and activity

To assess behavioural variation between sites, we measured individual-level boldness and activity in a subset of fish (*n* = 8 south, *n* = 7 north). In the context of this study, boldness was defined as the willingness to take risks to obtain food (e.g. striking prey in an unfamiliar space), while activity referred to the tendency to explore a novel environment. These behavioural axes represent components of animal temperament that can influence ecological interactions, including vulnerability to predation and capture via angling (Réale et al., 2007).

A shuttle box system (Loligo®) was used to evaluate these behaviours. The shuttle box consisted of two interconnected circular tanks linked by a small rectangular shuttle (total system length and width: 700 × 325 mm). Each tank was maintained at a constant water temperature of 12.5°C, with water recirculated between two aerated sumps at a flow rate of 240 ml∙min^−1^. Ambient air temperature was maintained at 9°C ± 0.5°C for the duration of the trial. Individual fish were randomly assigned to either the left or right tank and given a 1-hour acclimation period with no access to the adjoining chamber. After acclimation, a 1 × 5 cm strip of juvenile squid (*Sepioteuthis australis*, King Bait Berley & Tackle, Keysborough, VIC) with tentacles attached was tethered using monofilament line and a pyramid sinker and placed in the centre of the opposite tank. The barrier (shuttle) between the two tanks was then removed, providing the fish with visual and physical access to the bait.

Following removal of the shuttle, fish behaviour was recorded continuously for ~2 hours using ShuttleSoft 3.3 (Loligo Systems) and an ASUS C3 Full HD webcam (1920 × 1080 resolution, 30 fps). Lighting was adjusted to eliminate glare, and the system was enclosed in black opaque plastic to enhance tracking and reduce external visual disturbances for the fish. Videos were later analyzed for two primary behavioural metrics:

(i) **Time to strike bait,** used as an index of boldness; and.

(ii) **Time to enter the previously inaccessible tank,** used as a measure of exploratory activity.

### Statistical analyses

**Figure 3:**
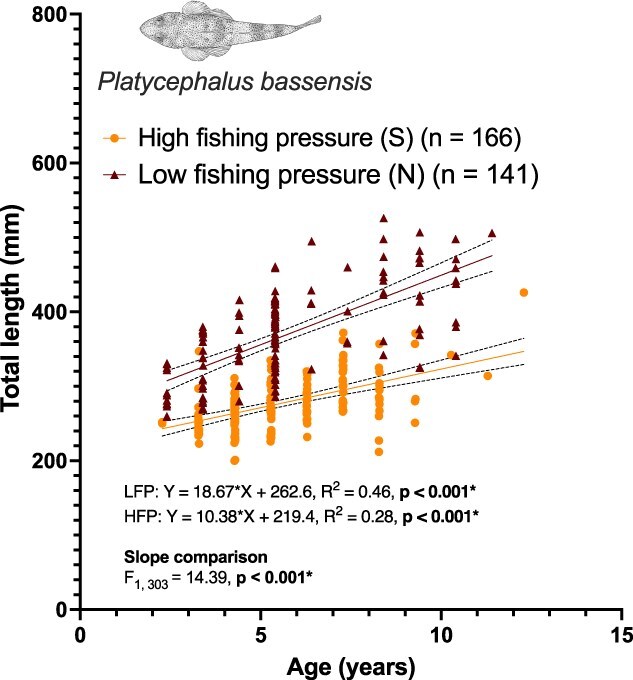
Relationship between total length (mm) and age (years) of sand flathead (*Platycephalus bassensis*) up to 13 years, sampled during the 2024 as part of IMAS Fisheries Independent Survey of recreational fisheries (https://tasfisheriesresearch.org/sfh/surveys/lf/). Fish were surveyed from Flinders Island (north, *n* = 141) and Frederick Henry Bay and the D’Entrecasteaux Channel (south, *n* = 166). Solid lines represent the best-fit regression lines for each region, with corresponding equations, *R*^2^ and *P*-values displayed on the graph. Dotted lines denote the 95% confidence intervals of the regression lines. An *F*-test comparing the slopes between regions, along with the associated *P*-value, is also shown. Statistical significance was determined at *P* < 0.05.

All statistical analyses were conducted in Prism v10.00 (GraphPad Software) and R v4.0.3 in RStudio. To test for regional differences in size-at-age, we primarily used age–length data from the 2024 IMAS Fisheries Independent Survey of recreational fisheries (https://tasfisheriesresearch.org/sfh/surveys/lf/), which provided larger sample sizes per site (Fig. 3). For comparison, the same analyses were also applied to our study subsample, and these results are presented in Supplementary Fig. S1. In both datasets, a linear model was fitted with total length as a function of age, region and their interaction (length − age × region). Age was treated as a continuous variable and fish older than 13 years were excluded, as they were only present in the northern sites. A linear model of length at age was selected over alternatives (e.g. Von Bertalanffy growth function) as size-at-age among the age range of fish present in the sample did not demonstrate slowing toward an asymptotic length and was approximately linear, confirmed by visual examination of diagnostic plots of model residuals. We also modelled integer age as a factor, in which case linear relationship between age and length was not assumed, but results were very similar (see supplementary Fig. S2; Supplementary Table S1). Metabolic rate (MR) did not differ between fish from Flinders Island and Musselroe Bay (*t*_22_ = 1.1, p = 0.3), so data from these sites were pooled. Two approaches were used to analyze MR across time post-capture. First, linear regression was used to assess the relationship between MR and days post-capture (MR ~ days), with model fit evaluated using *R*^2^ and the standard deviation of the residuals (*S_yx_*), and regional differences in the slope of this relationship was tested using an *F*-test. Second, MR across Weeks 1, 2 and 3 post-capture were analyzed using a repeated measures linear mixed-effects model with region and week as fixed effects and individual fish as a random effect (MR − region × week + (1 | fish)). Tukey's test was used for *post hoc* comparisons and the Geisser–Greenhouse correction (ε = 0.9470) was applied to account for potential violations of sphericity. To account for allometric effect of body size on metabolic rate, MR values were standardized (hereinafter MR_0.79_) to a common body mass (1 kg) using an a priori allometric scaling exponent of 0.79, the mean among teleost fishes reported by (Clarke and Johnston, 1999; Killen et al., 2010; Norin and Gamperl, 2018). The literature-derived exponent was used because the narrow size range of fish in the current study (<3-fold) limited the precision of fitting a scaling relationship for the species (White and Kearney, 2013). Analyses including body mass and age as covariates were performed using both linear (MR_0.79_ − region × day + age) and mixed-effects model (MR_0.79_ − region × day + age + (1|fish)) (Supplementary Table S2), and graphical relationships between MR and body size are provided in the Supplementary Information (Supplementary Fig. S3). Behavioural boldness was analyzed using Kaplan–Meier survival curves, with time to strike bait and enter a novel area treated as the event. Individuals that did not respond within 6000 seconds were right-censored. Significance was accepted at *P* < 0.05 and data are presented as mean ± standard error.

## Results

### Sand flathead from low fishing pressure regions were consistently larger at age

We found significant regional differences in age-length relationships between fish from the North and South of Tasmania. Fish total length increased with age in both regions, but the rates of change and predicted sizes at age differed markedly. The slope of the relationship between age and length was significantly steeper in fish from the North (18.67 ± 1.72 mm∙yr^−1^; *R*^2^ = 0.46, *S_yx_* = 46.98) than in the South (10.38 ± 1.29 mm∙yr^−1^; *R*^2^ = 0.28, *S_yx_* = 30.19; *F*_1,302_ = 14.39, *P* = 0.0002) (Fig. 3). When total length was analyzed with age as a factor, all ages above 2 years for which there were samples in both regions present, (i.e. ages <12 years) showed significant differences between regions (Supplementary Table S2) ranging between an average ± SEM of 62.29 ± 11.82 mm and 192.00 ± 53.57 mm.

Similar size-age patterns were also observed in the fish collected for this study, although significance was weaker as sample sizes were smaller (Supplementary Fig. S1). Length-at-age increased more steeply in northern fish (slope = 3.59 mm∙yr^−1^, *R*^2^ = 0.08, *P* = 0.024) than in southern fish (slope = 2.05 mm∙yr^−1^, *R*^2^ = 0.02, P = 0.28). Slopes did not differ significantly between regions (*F*₁,₁₃₃ = 0.30, *P* = 0.58), but intercepts differed markedly (*F*₁,₁₃₄ = 175.2, *P* < 0.0001), indicating that northern fish were consistently larger at a given age.

### Flathead from heavily fished regions exhibit elevated MR and greater post-capture metabolic reactivity

**Figure 4:**
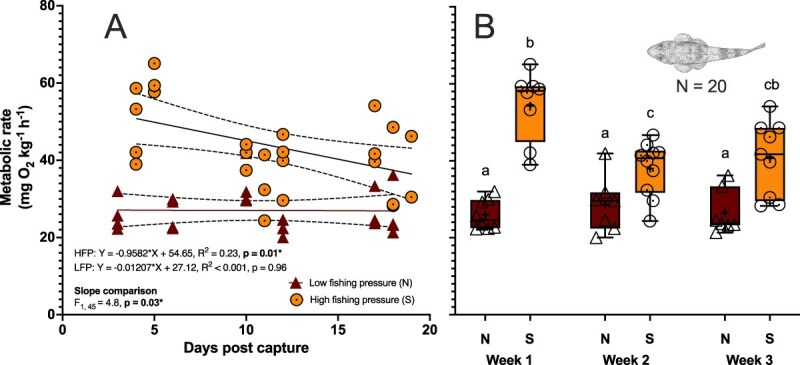
Relationship between standard MR and time post-catch for sand flathead (*Platycephalus bassensis*) from regions facing differential fishing pressure. (A) The relationship between MR (oxygen consumption in mg O_2_ kg^−1^∙h^−1^) and days post-catch for sand flathead sampled from regions of low fishing pressure (Flinders Island and Musselroe Bay; *n* = 8) and high fishing pressure (D’Entrecasteaux Channel; *n* = 12). The solid line represents the regression line, with the equation, *R*^2^, and *P*-value for the slope shown on the plot for each region. The dotted lines indicate the 95% confidence interval for the regression line. An *F*-test and corresponding *P*-value for the comparison of slopes between regions are also included. Data points represent individual fish responses from repeated measures. (B) MR grouped by week post-catch (weeks 1, 2 and 3), with comparisons between regions. Different letters (abc) above groups indicate statistically significant differences based on a mixed-effects analysis with REML estimation and Tukey’s multiple comparison post-hoc tests. Data are presented as a five number summary showing, min, max, median (−), mean (+), upper and lower quartile. Points represent values from individuals.

Linear regression was performed to evaluate the relationship between MR (mg O^2^ kg^−1^∙h^−1^) and days post-catch for fish caught from southern and northern regions (Fig. 4A). The slopes of the regression line between days post-catch and MR were found to significantly differ between northern and southern regions (*F*_1,45_ = 4.771, *P* = 0.03). For the southern fish, the slope was significantly non-zero (slope = −0.9582, 95% CI = −1.679 to −0.2377, *P* = 0.01), demonstrating a pronounced decline in oxygen consumption over days post-catch. In contrast, the northern fish had a slope that was not significantly different from zero (slope = −0.01207, 95% CI = −0.4735 to 0.4493, *P* = 0.9570), indicating no meaningful change in oxygen consumption over the same period. No relationship between MR and body size was observed in the fish sampled for measures of MR (south: *F*_1,9_ = 2.74, *P* = 0.13; north: *F*_1,6_ = 1.83, *P* = 0.2; pooled (north +south): *F*_1,17_ = 3.8, *P* = 0.07).

A repeated-measures mixed-effects model was used to examine the effects of region (north vs. south), week post-capture (Weeks 1–3), and their interaction on MR (Fig. 4B). There was a significant region × week interaction (*F*₂,₄₃ = 6.283, *P* = 0.004), indicating that the pattern of MR over time differed between regions. MR declined significantly over time in southern fish (*F*₁._894_,₄₀._7_₂ = 4.565, *P* = 0.018), whereas no clear change was observed in the northern fish. *Post hoc* comparisons confirmed a significant reduction in MR between Week 1 and Week 2 in the southern region (*P* = 0.01), but no significant differences between other time points. Southern fish also showed a 62% higher overall MR (mean = 44.3 mg O₂ kg^−1^ h^−1^) compared to northern fish (mean = 27.3 mg O₂ kg^−1^∙h^−1^), with a mean difference of −17.1 mg O₂ kg^−1^∙h^−1^ (95% CI: −21.5 to −12.6). Overall, even after accounting for the interaction with acclimation time, the effect of region was still significant (*F*_1, 43_ = 60.66, *P* < 0.0001), with oxygen consumption being higher in the south region compared to the north region over the studied period (up to 23 days post-catch). Pair-wise comparisons showed significant differences between southern and northern regions in all 3 weeks (W1 at *P* < 0.0001, W2 at *P* = 0.02 and W3 at *P* = 0.003, Fig. 4B).

For the final set of MR measurements in this study (Week 3, Days 21–23), mean MR in the south was 40.67 mg O₂ kg^−1^∙h^−1^, significantly higher than in the north (26.57 mg O₂ kg^−1^∙h^−1^; mean difference, −14.10; 95% CI, −22.45 to −5.75, *P* = 0.003). A similar pattern was observed in an independent dataset from a separate cohort of sand flathead assessed up to 54 days post-capture at the same test temperature (Rossiter-Hill et al. in prep), where MR remained elevated in the southern region, averaging 45.31 mg O₂ kg^−1^∙h^−1^ (n = 8), while the northern region maintained a lower average of 36.55 mg O₂ kg^−1^∙h^−1^ (95% CI, 26.46–46.65).

Analyses of allometrically corrected metabolic rate (MR_0.79_) supported these findings. Fixed-effects and mixed-effects analyses (MR₀·^79^ − region × day + age + (1|fish)) are summarized in Supplementary Table S2, and graphical relationships between MR_0.79_ and body mass are shown in Supplementary Fig. S3. Neither linear nor mixed-effects models detected an effect of age on MR_0.79_ (*t*₃₄ = −0.17, *P* = 0.87; *t*₁₁·_9_ = −0.27, *P* = 0.79).

### Boldness and exploratory behaviours did not differ between sand flathead from lightly fished and heavily fished regions

**Figure 5:**
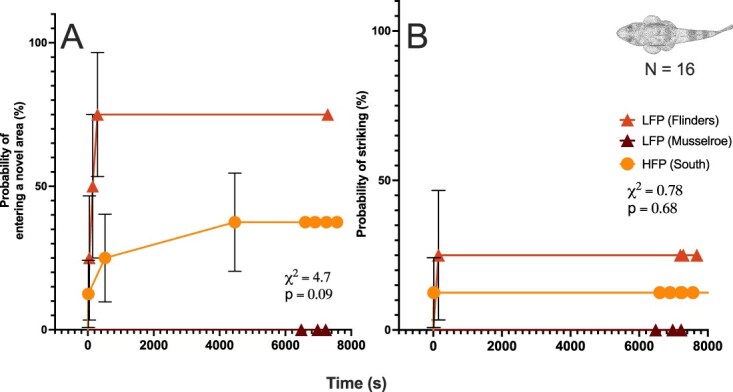
Behavioural responses of sand flathead (*Platycephalus bassensis*) from regions with high fishing pressure (D’Entrecasteaux Channel, *n* = 8) and low fishing pressure (Musselroe Bay, *n* = 3; Flinders Island, *n* = 4)**.** Time to event plots show probability of entering a novel area (%) (A) and probability of striking over time (%) (B). Each data point represents the cumulative proportion of individuals performing the behaviour at a given time, with vertical bars indicating the standard error (SE) of the estimate. Censored points are indicated at >6000 seconds. Statistical significance was assessed at *P* < 0.05 using a log-rank (Mantel-Cox) test (*X*^2^).

Time-to-event analysis revealed no statistically significant differences in exploratory behaviour (time to enter a novel area) among sand flathead from the two regions and sites within the northern region (log-rank test: χ^2^ = 4.708, df = 2, *P* = 0.095) (Fig. 5A). However, fish from Flinders Island (*n* = 4) were the most exploratory, with 75% (3/4) entering the novel area during the observation period, and a median entry time of 219.5 seconds (Fig. 5A). In contrast, none of the Musselroe fish (0/3) entered the novel area, and only three of eight fish (37.5%) from the southern region (D’Entrecasteaux Channel) did so. In both the southern and Musselroe region, the median response time was undefined because fewer than half of the individuals completed the behaviour. Censored data indicate the proportion of fish that did not perform the behaviour within the observation period (62.5% in the south, 25% in Flinders, and 100% in Musselroe).

Feeding behaviour (time to strike bait) also did not differ significantly between regions (log-rank test: χ^2^ = 0.778, df = 2, *P* = 0.678) (Fig. 5B). Only one individual in each of the Flinders (1/4; 25%) and southern (1/8; 12.5%) groups struck the bait, while no fish from Musselroe (0/3) did so. As with entry time, median strike times were undefined in all groups because fewer than 50% of individuals performed the behaviour. Censorship rates (i.e. fish that did not strike within the observation period) were therefore high: 87.5% in the south, 75% in Flinders, and 100% in Musselroe.

## Discussion

This study provides evidence of phenotypic divergence in sand flathead (*Platycephalus bassensis*) between regions with contrasting fishing pressure. Fish from the heavily fished southern region exhibited elevated MR, smaller size-at-age, and a transient post-capture elevation in MR relative to fish from the lightly fished north, but no detectable differences in boldness. Collectively, these results support our second hypothesis that high fishing mortality in the southern region may have favoured traits enabling rapid development and higher metabolism at the expense of prolonged somatic growth. Although correlative by nature, field comparisons, such as the one presented here, will be necessary for identifying potential signatures of selection in wild populations, where controlled manipulations are rarely feasible. In the sections that follow, we interpret these results in the context of life history theory, stress physiology, and fisheries-induced selection, while also considering alternative explanations including environmental variation, density dependence and sampling effects.

Life history theory predicts that high adult mortality favours earlier maturation and reduced investment in somatic growth, leading to smaller size at age and maturity (Roff, 2001; Jørgensen et al., 2007; Audzijonyte et al., 2016). Experimental harvest studies show similar patterns, with fast growing genotypes removed more often than slow growing ones, which accelerates these life history shifts in exploited populations (Conover and Munch, 2002; Biro and Post, 2008; Auer et al., 2018). In the D’Entrecasteaux Channel, adult fishing mortality exceeds natural mortality by more than fivefold and removes most adult females (Krueck et al., 2023). Under such intense and size-selective harvest, populations are therefore expected to become dominated by smaller earlier maturing phenotypes. Importantly, these shifts can arise through immediate demographic filtering of other life-history strategies, rather than genetic change. The selective removal of faster-growing individuals can bias observed size-at-age toward slower growing survivors, a demographic process known as the Rosa Lee effect (Lee, 1912; Kraak et al., 2019). In heavily fished systems, this demographic truncation is likely to account for much of the observed reduction in size at age, with any evolutionary contribution expected to be secondary, though not absent (Laugen et al., 2014). Disentangling these mechanisms would require back-calculated otolith growth and reproductive data to determine whether southern fish follow fundamentally different growth trajectories or simply represent a truncated size distribution.

Regardless of whether the removal of fast growing late maturing individuals is caused by direct (Rosa Lee) or indirect (evolutionary) effects, earlier maturation is expected to covary with higher metabolic rates (Pettersen et al., 2016; Auer et al., 2018). This prediction aligns well with our observations. Southern fish were smaller at age and showed higher MR than northern conspecifics. These patterns likely reflect the demographic consequences of intense harvest, but may also indicate a broader life-history shift toward a faster pace of life under high mortality. Nonetheless, our data remain correlative, as population-level replication was limited (Marshall, 2024) and environmental factors cannot be ruled out (Crespel et al., 2021). For example, intense harvest also alters density, prey availability, and competition, all of which affect growth and metabolism (Crespel et al., 2021). Typically, reduced density promotes faster growth and larger size-at-age, which would tend to mask rather than amplify the demographic or evolutionary signals of fishing (Audzijonyte et al., 2016; Morrongiello et al., 2019). This makes density-dependent effects an unlikely explanation for the smaller size-at-age and elevated metabolic rates observed in the south, but the effect of other environmental and random processes cannot strictly be excluded in the current experimental design. As Marshall (2024) noted, comparing two populations shows that they differ but not why they differ. Without replication across multiple independent contrasts, fishing and environmental effects remain confounded. Extending this framework to include multiple populations, ideally across a gradient of harvest intensity would enable stronger inference about causation but remains logistically challenging in this system. Sand flathead occupy discrete embayment’s separated by depth and coastline, shows strong site fidelity, and is heavily targeted across most of its range (Tracey et al., 2020; Krueck et al., 2024). No true unfished populations exist, and the ethical and logistical demands of capturing, transporting, and maintaining benthic fish across sites are substantial. These realities constrain replication but remain essential context for interpreting this work.

Both study regions fall within Tasmania's temperate eastern bioregion and experience broadly similar mean range of surface temperatures (Fig. 1) shaped by the same East Australian Current system (Ridgway, 2007; Ridgway and Ling, 2023). While small differences in thermal history can influence metabolic rate through acclimation, studies in fish show that metabolic compensation typically stabilizes within weeks, even following large temperature differences (Sandblom et al., 2014; Seebacher et al., 2015; Norin and Clark, 2016; Scheuffele et al., 2021). Given the relatively modest thermal differences among sites and the shared seasonal temperature range, any residual thermal effects on mass-specific metabolic rate are likely to be small. Consistent with this interpretation, an independent dataset from the same cohort measured up to 54 days post-capture under identical test conditions showed the same regional pattern, with southern fish maintaining consistently higher MR than northern conspecifics (Rossiter-Hill et al., in prep.). The persistence of this difference across cohorts supports the interpretation that it represents a stable physiological divergence rather than a short-term thermal artefact, though full developmental or trans-generational effects cannot be ruled out. For example, hatchery-reared lake sturgeon exhibited altered metabolic plasticity later in life depending on their rearing temperature, despite identical acute testing conditions (Genz and West, 2025). Similar multi-generational thermal carryover effects have been reported in other fishes, suggesting that developmental temperature can influence baseline metabolic rate (Pettersen et al., 2024). We therefore cannot exclude the possibility that long-term or inherited thermal histories have contributed to the divergence observed here. When it comes to other influences of environment, both regions share similar inner-shelf habitats of sand and mixed sand–seagrass that support nearly identical assemblages of small fishes and crustaceans (Ayling et al., 1975; Jordan, 2001). While minor differences in local productivity cannot be excluded, there is little evidence that such variation could account for the scale or consistency of the metabolic divergence observed.

Contrary to expectations from actively foraging species, boldness did not differ between regions and therefore does not seem to support the ‘timidity syndrome’ model proposed for actively foraging species, in which bold, high-MR individuals are preferentially captured and removed (Arlinghaus et al., 2017). In pelagic or more mobile taxa, boldness and metabolic rate are often linked to capture vulnerability (Biro and Stamps, 2010; Härkönen et al. (2014); Metcalfe et al., 2016). However, sand flathead are a sedentary, benthic ambush predatory, with high site fidelity (Tracey et al., 2020). Their behaviour is dominated by prolonged inactivity and cryptic posturing, behaviours which may not be well captured by open-field or novel area assays developed for more exploratory species. It therefore remains possible that behavioural variation exists but was not expressed under the current assay, particularly considering the modest sample sizes. Future work may wish to consider behavioural assays that include recovery from disturbance, refuge emergence or response to threat paradigms. Alternatively, the absence of detectable regional differences in boldness despite strong physiological divergence may suggest that selection in this system may act on metabolic or stress related traits rather than behavioural syndromes. Indeed, stress-related traits have been shown to better predict vulnerability to passive gears in other species (Louison et al., 2017).

Fish from the heavily fished south exhibited a pronounced, short term elevation in MR following capture that declined across subsequent weeks, whereas fish from the lightly fished north maintained low, stable MR throughout the same period. Because field baselines were not measured, we cannot confirm whether Week 1 values represent an elevation above natural metabolic levels. However, we interpret this pattern as a relative post-capture response*,* reflecting stronger initial reactivity in southern fish compared to northern lightly fished conspecifics. Such patterns are consistent with a reactive metabolic phenotype, characterized by strong but reversible responses to acute disturbance (Careau et al., 2008; Killen et al., 2015). Studies in other species show that metabolic reactivity can covary with capture vulnerability and stress responsiveness. For example, in *Micropterus salmoides*, individuals selectively bred for high angling vulnerability show elevated standard and maximum MR, greater aerobic scope and slower recovery from exhaustive exercise than low vulnerability lines (Redpath et al., 2010). These traits promote short-term performance, but longer-term energetic costs. Sutter et al. (2012) further demonstrated that such high vulnerability males also exhibited heightened aggression, increased parental care, and higher reproductive output, illustrating how metabolic and behavioural traits can covary in ways that influence both performance and exposure to capture. Although sand flathead differs from *M. salmoides* in ecology and life history, the physiological pattern observed here (elevated MR, reduced size at age, and a transient but marked increase in MR post-catch) is broadly consistent with the hypothesis that recreational fishing may select for individuals with more reactive phenotypes. Because sand flathead is a sedentary, benthic ambush predator targeted primarily by passive gear, vulnerability is likely more strongly influenced by physiological traits such as metabolic reactivity or stress sensitivity than by exploratory behaviours. The fact that only southern fish exhibited this transient post-capture response, suggests that repeated exposure to capture or other disturbance may have shaped population level differences in stress physiology.

If the regional differences in MR observed here persist in natural settings, they may have implications for resilience to both harvest and environmental change. The temporal decline in MR observed in southern fish may reflect a form of post-capture metabolic adjustment that enhances short-term responsiveness under repeated disturbance but also imposes higher energetic costs. Elevated MR can facilitate rapid physiological and behavioural reactions to acute stress, as demonstrated in other species where individuals with higher MR recover or respond more vigorously to disturbance (Killen et al., 2011, 2012, 2015). For example, juvenile *Dicentrarchus labrax* with higher metabolic rates showed greater post-startle activity and risk-taking behaviour under hypoxia, indicating that metabolic demand can amplify responsiveness during environmental or physiological stress (Killen et al., 2012). Such heightened reactivity may improve short-term survival or escape potential but also increases energetic expenditure (Killen et al., 2012). In environments subject to sustained fishing pressure, individuals capable of mounting strong but reversible metabolic responses to acute stress may gain a short term advantage if capture and release occur frequently, allowing them to recover more effectively between stress events.

Notably, none of the fish captured for this study reached the minimum size limit for sand flathead (35 cm), meaning that repeated capture and release is likely the norm in the southern region. Recurrent exposure to handling and air exposure may therefore represent a repeated disturbance regime capable of eliciting strong but transient metabolic responses. In contrast, northern fish maintained low, stable MR throughout the same period, consistent with a more conservative energetic strategy that may reflect selection for efficiency in environments where disturbance is infrequent. These contrasting responses suggest that long-term differences in fishing intensity could shape not only growth trajectories but also physiological stress responsiveness, potentially driving divergence in energetic strategies across regions. Indeed, Louison et al. (2017) found that stress responsiveness, rather than boldness or metabolic phenotype, was the primary driver of angling vulnerability in largemouth bass, with individuals showing smaller cortisol increases after air exposure being more likely to be captured. While elevated MR can support greater responsiveness and activity, it also increases baseline energetic costs and may reduce tolerance to chronic stressors such as warming, food limitation or hypoxia (Killen et al., 2011; Norin et al., 2016; Lefevre et al., 2017). As Tasmanian coastal waters continue to warm rapidly (Ridgway, 2007; Hobday and Pecl, 2014; Ridgway and Ling, 2023), populations already shaped by high fishing pressure may face compounded energetic constraints, highlighting the need to examine whether sustained harvest selects for metabolic profiles that trade immediate reactivity for longer-term resilience (Duncan et al., 2019).

In conclusion, this study reports marked physiological differences between sand flathead from a heavily fished southern and a lightly fished northern region of Tasmania. Whether these differences reflect plastic acclimation, cumulative effects of sustained fishing pressure, environmental variation or stochastic processes remains uncertain, but the patterns observed here are consistent with trends reported in other exploited species, where fishing pressure has been associated with changes in traits linked to capture vulnerability (Redpath et al., 2010; Sutter et al., 2012; Arlinghaus et al., 2017). Disentangling cause from correlation will require future studies that incorporate greater population replication and approaches including common-garden experiments, reciprocal transplants and demographic or genetic reconstruction to assess the stability and heritability of metabolic traits under contrasting harvest regimes. While such work is logistically challenging for moderately sized wild fish, it is necessary to determine whether the differences reported here persist beyond short-term plasticity. More broadly, this study illustrates how physiological traits can provide insight into differences among wild fish populations exposed to contrasting fishing pressure. In coastal systems, such as Tasmania, where recreational harvest is intense and ocean temperatures are rising, incorporating physiological perspectives will be increasingly important for understanding how fish will respond to multiple, overlapping pressures that can affect energy use, growth, size and performance.

## Supplementary Material

Web_Material_coag001

## Data Availability

Data supporting this manuscript are available in the supplementary materials as a downloadable.xls file.

## References

[ref1] Arlinghaus R, Laskowski KL, Alós J, Klefoth T, Monk CT, Nakayama S, Schröder A (2017) Passive gear-induced timidity syndrome in wild fish populations and its potential ecological and managerial implications. Fish Fish 18: 360–373.

[ref2] Audzijonyte A, Fulton E, Haddon M, Helidoniotis F, Hobday AJ, Kuparinen A, Morrongiello J, Smith AD, Upston J, Waples RS (2016) Trends and management implications of human-influenced life-history changes in marine ectotherms. Fish Fish 17: 1005–1028.

[ref3] Audzijonyte A, Fulton EA, Kuparinen A (2015) The impacts of fish body size changes on stock recovery: a case study using an Australian marine ecosystem model. ICES J Mar Sci 72: 782–792.

[ref4] Audzijonyte A, Kuparinen A, Gorton R, Fulton EA (2013) Ecological consequences of body size decline in harvested fish species: positive feedback loops in trophic interactions amplify human impact. Biol Lett 9: 20121103. 23365151 10.1098/rsbl.2012.1103 PMC3639762

[ref5] Auer SK, Dick CA, Metcalfe NB, Reznick DN (2018) Metabolic rate evolves rapidly and in parallel with the pace of life history. Nat Commun 9: 14. 29295982 10.1038/s41467-017-02514-z PMC5750215

[ref6] Ayling GM, Wilson KC, Ratkowsky DA (1975) Sand flathead (*Platycephalus bassensis*), an indicator species for mercury pollution in Tasmanian waters. Mar Pollut Bull 6: 142–144.

[ref7] Binder TR, Wilson ADM, Wilson SM, Suski CD, Godin J-GJ, Cooke SJ (2016) Is there a pace-of-life syndrome linking boldness and metabolic capacity for locomotion in bluegill sunfish? Anim Behav 121: 175–183. 10.1016/j.anbehav.2016.09.006

[ref8] Biro PA, Post JR (2008) Rapid depletion of genotypes with fast growth and bold personality traits from harvested fish populations. Proc Natl Acad Sci U S A 105: 2919–2922. 18299567 10.1073/pnas.0708159105 PMC2268560

[ref76] Biro PA, Stamps JA (2010) Do consistent individual differences in metabolic rate promote consistent individual differences in behavior? Trends Ecol Evol 25: 653–659. 10.1016/j.tree.2010.08.003 20832898

[ref9] Careau V, Thomas D, Humphries MM, Réale D (2008) Energy metabolism and animal personality. Oikos 117: 641–653.

[ref78] Chabot D, Steffensen JF, Farrell AP (2016) The determination of standard metabolic rate in fishes. J Fish Biol 88: 81–121. 26768973 10.1111/jfb.12845

[ref10] Chabot D, Zhang Y, Farrell AP (2021) Valid oxygen uptake measurements: using high r2 values with good intentions can bias upward the determination of standard metabolic rate. J Fish Biol 98: 1206–1216. 33332581 10.1111/jfb.14650 PMC9291193

[ref11] Clark TD, Messmer V, Tobin AJ, Hoey AS, Pratchett MS (2017) Rising temperatures may drive fishing-induced selection of low-performance phenotypes. Sci Rep 7: 40571. 28094310 10.1038/srep40571 PMC5240134

[ref12] Clarke A, Johnston NM (1999) Scaling of metabolic rate with body mass and temperature in teleost fish. J Anim Ecol 68: 893–905. 10.1111/j.1365-2656.2010.01672.x 20180875

[ref13] Conover DO, Arnott SA, Walsh MR, Munch SB (2005) Darwinian fishery science: lessons from the Atlantic silverside (*Menidia menidia*). Can J Fish Aquat Sci 62: 730–737.

[ref14] Conover DO, Munch SB (2002) Sustaining fisheries yields over evolutionary time scales. Science (New York, NY) 297: 94–96. 10.1126/science.1074085 12098697

[ref75] Coulson PG, Black BA, Potter IC, Hall NG (2014) Sclerochronological studies reveal that patterns of otolith growth of adults of two co-occurring species of Platycephalidae are synchronised by water temperature variations. Mar Bio 161: 383–393.

[ref15] Crespel A, Schneider K, Miller T, Rácz A, Jacobs A, Lindström J, Elmer KR, Killen SS (2021) Genomic basis of fishing-associated selection varies with population density. Proc Nat Acad Sci 118: e2020833118. 34903645 10.1073/pnas.2020833118 PMC8713780

[ref16] Department of Natural Resources and Environment Tasmania (2023) Sand Flathead in Tasmania – What’s Happening in the Fishery?

[ref17] Duncan MI, Bates AE, James NC, Potts WM (2019) Exploitation may influence the climate resilience of fish populations through removing high performance metabolic phenotypes. Sci Rep 9: 11437. 31391481 10.1038/s41598-019-47395-y PMC6685998

[ref18] Fraser K, Hartmann K, Krueck N (2021) *TASMANIAN SCALEFISH FISHERY ASSESSMENT* 2019/20.

[ref19] Genz J, West C (2025) Effects of rearing temperature on growth, energy reserves, and thermal plasticity of juvenile lake sturgeon. Fish Physiol Biochem 51: 124. 40690051 10.1007/s10695-025-01540-9

[ref20] Goodrich HR, Wood CM, Wilson RW, Clark TD, Last KB, Wang T (2024) Specific dynamic action: the energy cost of digestion or growth? J Exp Biol 227: 1–9. 10.1242/jeb.246722 38533751

[ref21] Han KY, Brennan RS, Monk CT, Jentoft S, Helmerson C, Dierking J, Hüssy K, Kokubun ÉE, Fuss J, Krause-Kyora B et al. (2025) Genomic evidence for fisheries-induced evolution in eastern Baltic cod. Sci Adv 11: eadr9889. 40561031 10.1126/sciadv.adr9889 PMC12189938

[ref77] Härkönen L, Hyvärinen P, Paappanen J, Vainikka A (2014) Explorative behavior increases vulnerability to angling in hatchery-reared brown trout (*Salmo trutta*). *Can J Fish Aquat Sci* 71: 1900–1909. 10.1139/cjfas-2014-0221

[ref22] Heino M, Pauli BD, Dieckmann U (2015) Fisheries-induced evolution. Annu Rev Ecol Evol Syst 46: 461–480.

[ref23] Hessenauer J-M, Vokoun JC, Suski CD, Davis J, Jacobs R, O’Donnell E (2015) Differences in the metabolic rates of exploited and unexploited fish populations: a signature of recreational fisheries induced evolution? PloS One 10: e0128336. 26039091 10.1371/journal.pone.0128336 PMC4454643

[ref24] Hobday AJ, Pecl GT (2014) Identification of global marine hotspots: sentinels for change and vanguards for adaptation action. Rev Fish Biol Fish 24: 415–425.

[ref25] Hollins J, Thambithurai D, Koeck B, Crespel A, Bailey DM, Cooke SJ, Lindström J, Parsons KJ, Killen SS (2018) A physiological perspective on fisheries-induced evolution. Evol Appl 11: 561–576. 29875803 10.1111/eva.12597 PMC5978952

[ref74] Hyndes GA (1992) Influence of sectioning otoliths on marginal increment trends and age and growth estimates for the flathead Platycephalus speculator. Fish Bull 90: 276–284.

[ref26] IMAS (2020) Tasmanian Wild Fisheries Assessments:Southern Sand Flathead. https://tasfisheriesresearch.org/sfh/ [accessed 30 May 2024].

[ref27] Jordan A (2001) Reproductive biology, early life-history and settlement distribution of sand flathead (*Platycephalus bassensis*) in Tasmania. Mar Freshw Res 52: 589–601.

[ref28] Jørgensen C, Enberg K, Dunlop ES, Arlinghaus R, Boukal DS, Brander K, Ernande B, Gårdmark AG, Johnston F, Matsumura S et al. (2007) Ecology: managing evolving fish stocks. Science 318: 1247–1248. 18033868 10.1126/science.1148089

[ref29] Killen SS, Atkinson D, Glazier DS (2010) The intraspecific scaling of metabolic rate with body mass in fishes depends on lifestyle and temperature. Ecol Lett 13: 184–193. 20059525 10.1111/j.1461-0248.2009.01415.x

[ref30] Killen SS, Marras S, McKenzie DJ (2011) Fuel, fasting, fear: routine metabolic rate and food deprivation exert synergistic effects on risk-taking in individual juvenile European sea bass. J Anim Ecol 80: 1024–1033. 21790592 10.1111/j.1365-2656.2011.01844.x

[ref31] Killen SS, Marras S, Ryan MR, Domenici P, McKenzie DJ (2012) A relationship between metabolic rate and risk-taking behaviour is revealed during hypoxia in juvenile European sea bass. Funct Ecol 26: 134–143.

[ref32] Killen SS, Nati JJH, Suski CD (2015) Vulnerability of individual fish to capture by trawling is influenced by capacity for anaerobic metabolism. Proc R Soc B Biol Sci 282: 20150603. 10.1098/rspb.2015.0603 PMC4632608 26246542

[ref33] Klefoth T, Skov C, Kuparinen A, Arlinghaus R (2017) Toward a mechanistic understanding of vulnerability to hook-and-line fishing: boldness as the basic target of angling-induced selection. Evol Appl 10: 994–1006. 29151855 10.1111/eva.12504 PMC5680629

[ref34] Kleiber M (1947) Body size and metabolic rate. Physiol Rev 27: 511–541. 20267758 10.1152/physrev.1947.27.4.511

[ref35] Kraak SBM, Haase S, Minto C, Santos J (2019) The Rosa Lee phenomenon and its consequences for fisheries advice on changes in fishing mortality or gear selectivity. ICES J Mar Sci 76: 2179–2192.

[ref36] Krueck N, Marshell A, Coulson P, Sharples R, Cresswell K, Tracey S (2023) *Southern Sand Flathead Assessment* 2023.

[ref37] Krueck N, Marshell A, Coulson P, Sharples R, Cresswell K, Tracey S (2024) *Southern Sand Flathead Assessment* 2024.

[ref38] Laugen AT, Engelhard GH, Whitlock R, Arlinghaus R, Dankel DJ, Dunlop ES, Eikeset AM, Enberg K, Jørgensen C, Matsumura S et al. (2014) Evolutionary impact assessment: accounting for evolutionary consequences of fishing in an ecosystem approach to fisheries management. Fish Fish 15: 65–96. 10.1111/faf.12007 PMC4579828 26430388

[ref39] Lee RM (1912) An investigation into the methods of growth determination in fishes by means of scales. Journal du Conseil s1: 3–34.

[ref40] Lefevre S, McKenzie DJ, Nilsson GE (2017) Models projecting the fate of fish populations under climate change need to be based on valid physiological mechanisms. Glob Chang Biol 23: 3449–3459. 28168760 10.1111/gcb.13652

[ref41] Louison MJ, Adhikari S, Stein JA, Suski CD (2017) Hormonal responsiveness to stress is negatively associated with vulnerability to angling capture in fish. J Exp Biol 220: 2529–2535. 28724703 10.1242/jeb.150730

[ref42] Marshall DJ (2024) Principles of experimental design for ecology and evolution. Ecol Lett 27: e14400. 38591235 10.1111/ele.14400

[ref43] Matsumura S, Arlinghaus R, Dieckmann U (2011) Assessing Evolutionary Consequences of Size-selective Recreational Fishing on Multiple Life-history Traits, with an Application To Northern Pike (*Esox lucius*). Monograph, IR-11-009, IIASA, Laxenburg, Austria. https://pure.iiasa.ac.at/id/eprint/9825/, https://iiasa.dev.local/

[ref44] Metcalfe NB, Van Leeuwen TE, Killen SS (2016) Does individual variation in metabolic phenotype predict fish behaviour and performance? J Fish Biol 88: 298–321. 10.1111/jfb.12699. 26577442 PMC4991269

[ref45] Morrongiello JR, Sweetman PC, Thresher RE (2019) Fishing constrains phenotypic responses of marine fish to climate variability. J Anim Ecol 88: 1645–1656. 31034605 10.1111/1365-2656.12999

[ref46] Norin T, Clark TD (2016) Measurement and relevance of maximum metabolic rate in fishes. J Fish Biol 88: 122–151. 26586591 10.1111/jfb.12796

[ref47] Norin T, Gamperl AK (2018) Metabolic scaling of individuals vs. populations: evidence for variation in scaling exponents at different hierarchical levels. Funct Ecol 32: 379–388.

[ref48] Norin T, Malte H, Clark TD (2016) Differential plasticity of metabolic rate phenotypes in a tropical fish facing environmental change. Funct Ecol 30: 369–378.

[ref49] Pettersen AK, Metcalfe NB, Seebacher F (2024) Intergenerational plasticity aligns with temperature-dependent selection on offspring metabolic rates. Phil Trans R Soc B Biol Sci 379: 20220496. 10.1098/rstb.2022.0496 PMC10772613 38186279

[ref50] Pettersen AK, White CR, Marshall DJ (2016) Metabolic rate covaries with fitness and the pace of the life history in the field. Proc R Soc B Biol Sci 283: 20160323. 10.1098/rspb.2016.0323 PMC4892794 27226476

[ref51] Réale D, Reader SM, Sol D, McDougall PT, Dingemanse NJ (2007) Integrating animal temperament within ecology and evolution. Biol Rev Camb Philos Soc 82: 291–318. 17437562 10.1111/j.1469-185X.2007.00010.x

[ref52] Redpath T.D, Cooke S.J, Suski C.D, Arlinghaus R, Couture P, Wahl D.H, Philipp D.P (2010) The metabolic and biochemical basis of vulnerability to recreational angling after three generations of angling-induced selection in a teleost fish. Can J Fish Aquat Sci 67, 1983–1992.

[ref53] Ridgway KR (2007) Long-term trend and decadal variability of the southward penetration of the east Australian current. Geophys Res Lett 34: 1–5.

[ref54] Ridgway KR, Ling SD (2023) Three decades of variability and warming of nearshore waters around Tasmania. Prog Oceanogr 215: 103046.

[ref55] Roff D (2001) Life History, Evolution of. In SA Levin, ed, Encyclopedia of Biodiversity, EdSecond. Academic Press, Waltham, pp. 631–641

[ref56] Sandblom E, Gräns A, Axelsson M, Seth H (2014) Temperature acclimation rate of aerobic scope and feeding metabolism in fishes: implications in a thermally extreme future. Proc R Soc B Biol Sci 281: 20141490. 10.1098/rspb.2014.1490 PMC4211447 25232133

[ref57] Scheuffele H, Rubio-Gracia F, Clark TD (2021) Thermal performance curves for aerobic scope in a tropical fish (*Lates calcarifer*): flexible in amplitude but not breadth. J Exp Biol 224: jeb243504. 34821366 10.1242/jeb.243504

[ref58] Scrucca L, Fop M, Murphy TB, Raftery AE (2016) Mclust 5: clustering, classification and density estimation using Gaussian finite mixture models. The R Journal 8: 289. 27818791 PMC5096736

[ref59] Secor (2009) Specific dynamic action: a review of the postprandial metabolic response. J Comp Physiol B 179: 1–56. 18597096 10.1007/s00360-008-0283-7

[ref60] Seebacher F, White CR, Franklin CE (2015) Physiological plasticity increases resilience of ectothermic animals to climate change. Nat Clim Change 5: 61–66.

[ref61] Stamps JA (2007) Growth-mortality tradeoffs and ‘personality traits’ in animals. Ecol Lett 10: 355–363. 17498134 10.1111/j.1461-0248.2007.01034.x

[ref62] Sutter DAH, Suski CD, Philipp DP, Klefoth T, Wahl DH, Kersten P, Cooke SJ, Arlinghaus R (2012) Recreational fishing selectively captures individuals with the highest fitness potential. Proc Natl Acad Sci U S A 109: 20960–20965. 23213220 10.1073/pnas.1212536109 PMC3529059

[ref63] Svendsen M.B.S, Bushnell P, Steffensen JF (2019) AquaResp 3. Zenodo, Geneva, Switzerland. https://zenodo.org/records/2584015 [accessed 4 April 2025].

[ref64] Svendsen MBS, Bushnell PG, Steffensen JF (2016) Design and setup of intermittent-flow respirometry system for aquatic organisms. J Fish Biol 88: 26–50. 26603018 10.1111/jfb.12797

[ref65] Swain DP, Sinclair AF, Mark Hanson J (2007) Evolutionary response to size-selective mortality in an exploited fish population. Proc Biol Sci 274: 1015–1022. 17264058 10.1098/rspb.2006.0275 PMC2124474

[ref66] Tracey S, Stark K (2024) 2022/23 SURVEY OF RECREATIONAL FISHING IN TASMANIA. Institute for Marine and Antarctic Studies, Hobart Tasmania

[ref67] Tracey SR, Hartmann K, McAllister J, Lyle JM (2020) Home range, site fidelity and synchronous migrations of three co-occurring, morphologically distinct estuarine fish species. Sci Total Environ 713: 136629. 31955103 10.1016/j.scitotenv.2020.136629

[ref68] Tudorache C, Schaaf MJM, Slabbekoorn H (2013) Covariation between behaviour and physiology indicators of coping style in zebrafish (*Danio rerio*). J Endocrinol 219: 251–258. 24198397 10.1530/JOE-13-0225

[ref69] Väätäinen R, Huuskonen H, Hyvärinen P, Kekäläinen J, Kortet R, Torrellas Arnedo M, Vainikka A (2018) Do metabolic traits, vulnerability to angling, or capture method explain boldness variation in Eurasian Perch? Physiol Biochem Zool 91: 1115–1128. 30295572 10.1086/700434

[ref70] Waples RS, Audzijonyte A (2016) Fishery-induced evolution provides insights into adaptive responses of marine species to climate change. Front Ecol Environ 14: 217–224.

[ref71] White CR, Kearney MR (2013) Determinants of inter-specific variation in basal metabolic rate. J Comp Physiol B 183: 1–26. 23001691 10.1007/s00360-012-0676-5

[ref72] Wilson ADM, Binder TR, McGrath KP, Cooke SJ, Godin J-GJ (2011) Capture technique and fish personality: angling targets timid bluegill sunfish, *Lepomis macrochirus**.* Can J Fish Aquat Sci 68: 749–757.

[ref73] Yuan M, Chen Y, Huang Y, Lu W (2018) Behavioral and metabolic phenotype indicate personality in zebrafish (*Danio rerio*). Front Physiol 9: 1–10. 29899710 10.3389/fphys.2018.00653 PMC5988878

