# Supplementary material for: Phenotypic divergence of sand flathead (Platycephalus bassensis) between heavily and lightly fished regions in Tasmania, Australia
Source: Conserv Physiol. 2026 Feb 5;14(1):coag001. doi: 10.1093/conphys/coag001 (PMC12885885; doi:10.1093/conphys/coag001)
Supplement: Web_Material_coag001 [file web_material_coag001.zip › Supplementary information for Phenotypic divergence of sand flathead.pdf]

Supplementary information for Phenotypic divergence of sand flathead (*Platycephalus bassensis*) between heavily and lightly fished regions in Tasmania, Australia

Harriet R Goodrich[1], Finlay Rossiter-Hill[1], Asta Audzijonyte, Barrett W Wolfe[1] , Rachel Breslin[1], Sean R Tracey[1]

1. Institute for Marine and Antarctic Studies, University of Tasmania, Private Bag 49, Hobart, TAS 7001, Australia

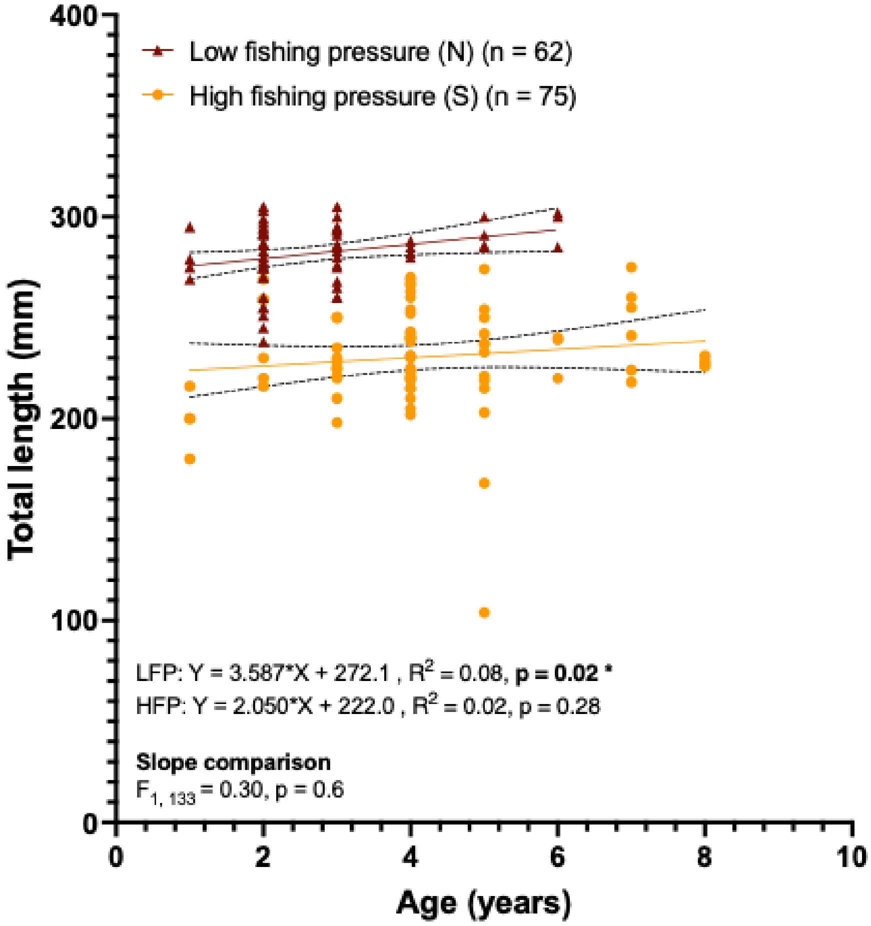

Supplementary Figure S1: Relationship between total length (mm) and age (years) of sand flathead (*Platycephalus bassensis*) used as experimental animals in this study. Fish were sampled from low fishing pressure sites in the north (Flinders Island, Musselroe Bay, and Eddystone Point; n = 62) and from a high fishing pressure site in the south (D'Entrecasteaux Channel; n = 75). Solid lines represent the best-fit regression lines for each region, with

corresponding equations, R^2 and p-values displayed on the graph. Dotted lines denote the 95% confidence intervals of the regression lines. F-tests comparing slopes between regions, along with associated p-values, are also shown. Statistical significance was determined at $p < 0.05$.

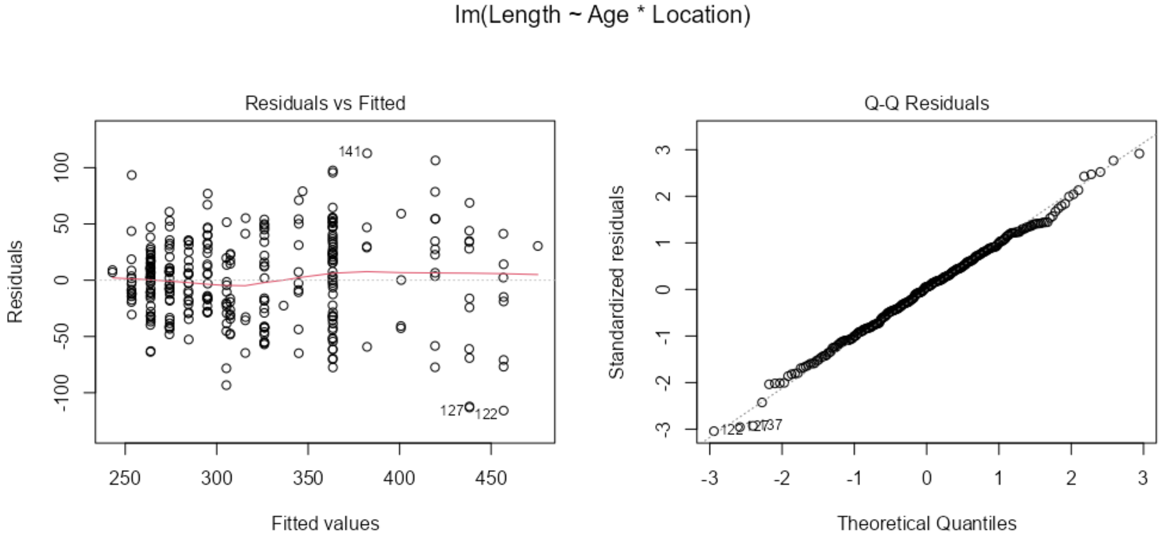

Supplementary Figure S2: Residual plots of the total length ~ age × region model for sand flathead (*Platycephalus bassensis*), showing residuals versus fitted values (left) and Q–Q residuals (right). The absence of strong patterns in the residuals and approximate normality support the use of a linear model rather than a non-linear growth function.

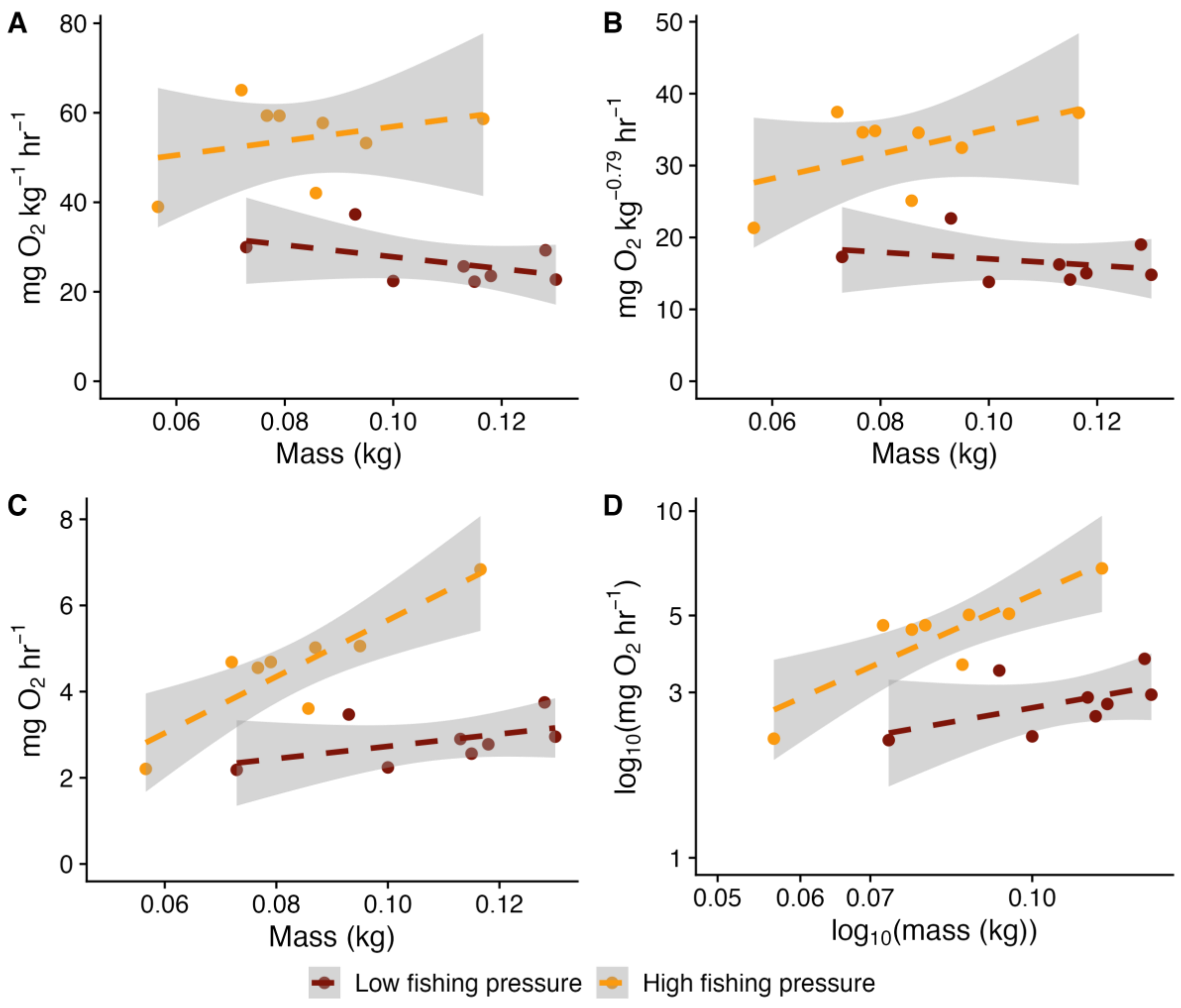

Supplementary Figure S3. Oxygen consumption during the first week post-capture for sand flathead (*Platycephalus bassensis*) from regions with contrasting fishing pressure, plotted against body mass (kg). (A) Mass-specific metabolic rate (mg O_2 kg^{-1} h^{-1}). (B) Allometrically scaled metabolic rate (mg O_2 $kg^{-0.79}$ h^{-1}) using the mass-scaling exponent of 0.79 from Clarke and Johnston (1999). (C) Absolute oxygen consumption (mg O_2 h^{-1}) on original, and (D) log–log axes. Dashed lines show linear regressions for each region (North (red lines and points): n = 8, low fishing pressure; South (yellow line and points): n = 8, high fishing pressure), with shaded areas indicating 95% confidence intervals.

Supplementary Table 1: Least-squares means contrasts from the total length model for sand flathead 2024 IMAS Fisheries Independent Survey of recreational fisheries (https://tasfisheriesresearch.org/sfh/surveys/lf/), (*Platycephalus bassensis*), with age treated as a categorical factor (length ~ age × region). The table reports pairwise comparisons of mean total length between northern (Flinders Island, n = 141) and southern (Frederick Henry Bay

and D'Entrecasteaux Channel, n = 166) regions within each age class, adjusted for multiple comparisons. Reported statistics include age class, estimated length difference (mm), standard error (SE) of the estimate, degrees of freedom (df), t-ratio, and p-value (significance highlighted in bold)

Age	Length difference (mm)	SE	df	t.ratio	p
2	41.75	28.93	286	1.44	0.15
3	62.29	11.82	286	5.27	**0.00**
4	90.37	13.14	286	6.87	**0.00**
5	95.48	8.57	286	11.15	**0.00**
6	130.43	18.85	286	6.92	**0.00**
7	88.04	20.46	286	4.30	**0.00**
8	157.17	15.26	286	10.30	**0.00**
9	112.78	20.43	286	5.52	**0.00**
10	84.87	40.18	286	2.11	**0.04**
11	192.00	53.57	286	3.58	**0.00**

Supplementary Table S2: Summary of fixed effects from the linear regression and mixed-effects models examining variation in standard metabolic rate (SMR; mg O_2 kg^{-1} h^{-1}) of *Platycephalus bassensis* with region, week, days post catch, and age. Analyses were performed on SMR values allometrically corrected to a 0.79 mass-scaling exponent. Significant effects highlighted in bold.

Model	*Fixed effects*	*Estimate (± SE)*	*F / t value*	*df*	*p-value*
Regression *($MR_{0.79}$ ~ region * day + age)*	Intercept	17.9969 ± 3.03	t = 5.94	34	**< 0.001**
	Region (south)	+16.7339 ± 4.46	t = 3.75	34	**0.001**
	Day	−0.0584 ± 0.23	t = −0.26	34	0.80
	Age	−0.1059 ± 0.63	t = −0.17	34	0.87
	Region × Day	−0.5107 ± 0.32	t = −1.57	34	0.12
ANOVA (Type III)	Region	408.6	F = 14.1	1, 34	**0.001**
	Day	1.9	F = 0.07	1, 34	0.80

	Age	0.8	F = 0.03	1, 34	0.87
	Region × Day (slope comparison)	72.0	F = 2.48	1, 34	0.12
Mixed-effects model *($MR_{0.79}$ ~ region * week + age + (1| fish))*	Intercept	17.31 ± 1.92	t = 9.01	25.9	**< 0.001**
	Region (south)	+18.83 ± 2.98	t = 6.32	27.3	**< 0.001**
	Week 2	+0.58 ± 2.28	t = 0.25	19.8	0.80
	Week 3	+0.17 ± 2.30	t = 0.07	22.5	0.94
	Age	−0.15 ± 0.54	t = −0.27	11.9	0.79
	Region × Week 2	−13.66 ± 3.19	t = −4.28	20.1	**< 0.001**
	Region × Week 3	−7.91 ± 3.34	t = −2.37	22.9	**0.027**
